# The fall in income inequality during COVID-19 in four European countries

**DOI:** 10.1007/s10888-021-09499-2

**Published:** 2021-08-08

**Authors:** Andrew E. Clark, Conchita D’Ambrosio, Anthony Lepinteur

**Affiliations:** 1 Paris School of Economics - CNRS, Paris, France; 2 University of Luxembourg, Esch-sur-Alzette, Luxembourg

**Keywords:** COME-HERE, COVID-19, Income inequality, D63, I32, I38

## Abstract

We here use panel data from the COME-HERE survey to track income inequality during COVID-19 in France, Germany, Italy, and Spain. Relative inequality in equivalent household disposable income among individuals changed in a hump-shaped way between January 2020 and January 2021, with an initial rise from January to May 2020 being more than reversed by September 2020. Absolute inequality also fell over this period. Due to the pandemic some households lost more than others, and government compensation schemes were targeted towards the poorest, implying that on average income differences decreased. Generalized Lorenz domination reveals that these distributive changes reduced welfare in Italy.

**Supplementary Information:**

The online version contains supplementary material available at 10.1007/s10888-021-09499-2.

**JEL Classification** D63 · I32 · I38

We would like to thank Thomas Blanchet, Liyousew Borga, Walter Bossert, Michael Förster, Markus Grabka, Sebastian Königs, Giorgia Menta, Vincent Vergnat, Remi Yin and participants at the OECD WISE Webinar Series on COVID-19 and Inequality and a seminar at the University of Luxembourg for their help and feedback. We also thank the Editor, Andreas Peichl, and two anonymous referees for comments that helped improve the paper. Financial support from the French Agence Nationale de la Recherche (DYNPANDEMIC), André Losch Fondation, Art2Cure, Cargolux, and the Fonds National de la Recherche Luxembourg (Grant COVID-19/2020-2/14840950/COME-HERE) is gratefully acknowledged. Andrew Clark acknowledges financial support from the EUR grant ANR-17-EURE-0001.

✉ Conchita D'Ambrosio
conchita.dambrosio@uni.lu

Andrew E. Clark
andrew.clark@ens.fr

Anthony Lepinteur
anthony.lepinteur@uni.lu

## 1 Introduction

At the time of writing, over 150,000,000 cases of COVID-19 have been reported globally, and the number of new infections in some Western European countries like France, Germany, Italy and Spain is just starting to fall thanks to vaccination campaigns. In line with epidemiological models (Ferguson et al. 2020; Lourenco et al. 2020), many governments adopted policies starting in March 2020 that aimed to restrict population movement (such as lockdowns, travel restrictions and curfews). The rationale for these restrictions was to save lives and prevent health systems from being overwhelmed. These restrictive policies have produced unprecedented effects on household incomes, which governments have addressed via extraordinary measures such as furlough payments and the direct support targeted at those who were more in need during the pandemic. While much of this effort has been national, in Europe the European Union recently agreed to complement national programmes via the largest-ever stimulus package of €1.8 Trillion to help rebuild a greener, more-digital and more-resilient post-COVID-19 Europe.

There is a very fast-growing literature on the impact of COVID-19 lockdowns (some examples are Brodeur et al. 2021, Layard et al. 2020, and Fang et al. 2020), and more generally the economic and distributional consequences of the COVID-19 pandemic. Our aim here is to track household disposable-income inequality during the COVID-19 period using direct information from surveys on income.

We have access to a unique longitudinal high-frequency information on household disposable income in four European countries (France, Germany, Italy and Spain) from the COME-HERE panel survey run by the University of Luxembourg since the end of April 2020. COME-HERE allows us to track relative and absolute inequality in equivalent disposable household income among individuals from January 2020, our pre-COVID-19 observation, to January 2021.

In line with the predictions from most micro-simulation analysis (Almeida et al. 2021, Brewer and Tasseva 2021, O'Donoghue et al. 2020, Li et al. 2020, Brunori et al. 2020), relative inequality in our four countries fell from January 2020 to January 2021. The same country pattern is found in indices of absolute inequality (measuring the gaps in income levels, as opposed to shares). Following the pandemic, some households lost more than others, as we will discuss in detail below. Since government compensation schemes were both targeted towards the poorest and transferred amounts that mostly did not fully compensate for losses of the richer, income differences decreased.

The decomposition of these changes in relative inequality by age, gender, education, and labour-force and marital statuses reveals that most movement has been within groups; the between-group changes are mixed.

The remainder of the paper is structured as follows. Section 2 reviews the literature on the effect of the COVID-19 pandemic on income changes and inequality, and Section 3 describes the COME-HERE survey and the measures of inequality we use. Section 4 then presents our results regarding the evolution of income inequality in France, Germany, Italy and Spain between January 2020 and January 2021. Section 5 presents a number of robustness checks, and Section 6 concludes.

## 2 Literature review

The COVID-19 pandemic has had substantial effects on individual outcomes. Some of the work in this area has explicitly focused on the labour market. Adams-Prassl et al. (2020) show that the pandemic in March and April 2020 had a negative impact on labour-force participation (LFP) and working time: these effects were stronger in the UK and the US than in Germany, and, within countries, hit less-educated workers and women harder, and so exacerbated pre-existing inequality. Using US data from the American Time Use Survey in 2017 and 2018, Alon et al. (2021) predict that the COVID-19 shock will increase gender inequality by placing a disproportionate burden on women. Compared to past recessions, the fall in employment due to social distancing had a greater effect on sectors with high female-employment rates; at the same time, women have shouldered the lion's share of the burden of greater childcare following the closure of schools and daycare centres. Using Spanish data, Farré et al. (2020) come to similar conclusions. Exploiting variations across US States in COVID-19 cases and death, Beland et al. (2020) show that COVID-19 increased unemployment, reduced hours of work and LFP, but had no significant effect on wages: these detrimental effects were more pronounced for some types of workers (e.g., Hispanic and younger workers). Similar conclusions are reached by Guven et al. (2020), using the Longitudinal Labour Force Survey data up to the end of May 2020 in Australia: COVID-19 reduced LFP by 2.1 %, increased unemployment by 1.1 % and produced a one-hour drop in weekly working hours. The effect again differed across groups, with the LFP and working hours of the less-educated being more affected, and unemployment rising more for immigrants and those with shorter job tenure or occupations unsuitable for remote work. Bottan et al. (2020) equally underline the greater impact of COVID-19 on the least well-off in Latin America and the Caribbean between January and April 2020 using online-survey data. Bonacini et al. (2020) simulate the feasibility of working from home using data on worker characteristics in Italian surveys from 2013 to 2018. Working from home is suggested to be easier for male, older, better-educated and higher-paid workers, increasing labour-income inequality. The four hypothetical scenarios of stringent policy response across 29 European countries in Palomino et al. (2020) also produce uneven wage losses and rising wage inequality.

The above contributions covered the labour market; we here wish to focus on household disposable income, of which labour income is only one (important) part. Beyond direct labour-market intervention, governments have also implemented a variety of other policies, such as mortgage holidays, rent support, and fiscal, monetary and macro-financial policies. The policy tracker of the IMF contains an excellent, and regularly-updated, summary of the key government economic responses in 197 countries (see https://www.imf.org/en/Topics/imf-and-covid19/Policy-Responses-to-COVID-19).

Two broad approaches have been taken to track household disposable-income inequality during COVID-19. The first appeals to micro-simulation and calibration, due to the scarcity of adequate recent micro data. Using EUROMOD, Almeida et al. (2021) simulate separately the effect of the pandemic and the subsequent policy responses in 27 European countries. In the absence of policy response, the 2020 relative Gini coefficient would have risen by 3.6 %, but following the policy response relative inequality it instead fell by 0.7 %. Brewer and Tasseva (2021) reach the same conclusion using the UK module of EUROMOD, with there being lower values of the Gini coefficient, Theil index and Mean Logarithmic Deviation in 2020 from the COVID-19 policy responses and the pre-existing tax-benefits system; see also O'Donoghue et al. (2020) and Li et al. (2020) for Ireland and Australia, respectively. Brunori et al. (2020) simulate the short-term effects of two months of lockdown on the Italian income distribution in the IRPET MicroReg tax model. Again, the Gini coefficient for equivalent disposable household income falls due to policy interventions that target the poorest, from 0.3396 to 0.3373. However, the

Italian analysis using EUROMOD in Figari and Fiorio (2020) predicts rising inequality from one month of lockdown.

The second approach to income inequality uses direct information from surveys on income changes. This data is scarcer, and is often cross-sectional and of relatively-low frequency. Brewer and Gardiner (2020) suggest that the probability of reporting lower household income was relatively constant across pre-COVID-19 income quintiles in a cross-section of 6,000 UK adults in early May 2020. Belot et al. (2021) use cross-section data from China, Japan, South Korea, Italy, the UK and the US in April 2020 (around 1,000 respondents per country) to show that those aged 18–25 were more likely to experience a drop in household income. Neither of these papers calculates formal inequality indices. Very recently, Grabka (2021) analysed the COVID-19 sample of the German Socio-Economic Panel (SOEP), and showed that the Gini coefficient on current net monthly household income in Germany was slightly lower during the second lockdown of January/February 2021 as compared to its 2019 value. This relative inequality decline is attributed to the income losses suffered by the self-employed, with downwards movements from the top half of the income distribution. Grabka (2021) also reports no change in the average income of those in the bottom half of the income distribution thanks to the Federal government's expansive economic policy. Our results for Germany confirm all of the above findings, and generalise them to other EU countries.

The scarcity of data from surveys on income, let alone longitudinal and cross-country, is understandable given the cost and associated challenges. As Figari and Fiorio (2020, p.2) note: "*Lack of longitudinal up-to-date information on household income and labour market circumstances, usually available a few years after the economic shock and in a limited number of countries only, constrains the possibilities for empirical analysis*". Our use of recent, high-frequency cross-country panel data helps to fill this gap.

## 3 Data and method

The data we use here are from the COME-HERE (COVID-19, MEntal HEalth, REsilience and Self-regulation) panel survey collected by the University of Luxembourg. The survey was conducted by Qualtrics in France, Germany, Italy, Spain and Sweden. Respondents complete an on-line questionnaire that takes approximately 20 min. Qualtrics runs specialised recruitment campaigns via its partner network, and is able to contact groups that may be hard to reach on the internet (older respondents, for example). Qualtrics uses stratified sampling, and the COME-HERE samples are nationally-representative in terms of age, gender and region of residence. Qualtrics also has data-quality protocols: for instance, the information supplied by respondents who answer the questionnaire in under ½ of the median survey-completion time is not retained, and a replacement interview is conducted. In the same spirit, the IP addresses of the respondents are checked and digital-fingerprinting technology is used to ensure that observations are not duplicated. Ethics approval for our study was granted by the Ethics Review Panel of the University of Luxembourg. The COME-HERE dataset collects information at the individual and household levels, and is longitudinal. The first five waves of survey, which provide the data analysed here, were conducted around May 1st 2020, June 9th 2020, September 5th 2020, November 20th 2020 and March 1st 2021. At least three more waves are planned for 2021.

Over 8,000 individuals took part in the first survey wave, and were then invited to respond to all subsequent waves (there have been no refreshment samples, given the satisfactory response rates). Around 85 % took part in at least one other survey wave, with 39.3 % participating in all five waves, 22.2 % in four waves, 12.1 % in three waves, and 11 % in two waves. Our analysis will be carried out on unbalanced panel data. We do not restrict our analysis to the balanced panel as this would sharply reduce the total number of observations per country (with under 40 % of respondents participating in all five surveys), and it is traditional in the inequality literature not to do so. The balanced panel actually produces figures, available upon request, that are somewhat in line with those from the unbalanced panel. Moreover, we show in the robustness checks that the use of sample weights, which guarantee national representativeness and account for selective attrition, makes little difference to our results.

The objective of the survey is to not only collect sufficient individual information to describe living and mental-health conditions during COVID-19, but also identify recent changes and events that might have affected individuals' lives. Standard sociodemographic characteristics such as age, gender, education, and labour-force and marital statuses were also collected. Special survey modules in some waves addressed topics such as risk attitudes, time discounting, preferences for redistribution, income comparisons, and working conditions.

We measure income inequality via a question in each survey wave asking respondents about their household disposable income two to four months prior to the survey, with responses in the following bands: "*0 to 1250 Euros*", "*1250 to 2000 Euros*", "*2000 to 4000 Euros*", "*4000 to 6000 Euros*", "*6000 to 8000 Euros*", "*8000 to 12500 Euros*" and "*Over 12500 Euros*". Our empirical analysis will cover household disposable income at four points in time. The first is January 2020 (reported in Wave 1), that we will take as the pre-COVID-19 figure. The second refers to May 2020 (from Wave 3), at the end of the first COVID-19 wave. The third refers to September 2020 (from Wave 4), after the Summer but before the beginning of the second wave of COVID-19 in Europe, while the fourth point in time is January 2021 during the third COVID-19 wave. The income question in Wave 2 adds little information, as it comes from April 2020, yielding very similar figures to those for May 2020 from Wave 3.

To track the evolution of income inequality across Europe, we first estimate Lorenz curves and calculate four relative measures of inequality: Gini, and three members of the Generalized Entropy family - Mean Logarithmic Deviation (GE(0)), Theil (GE(1)) and half the square of the Coefficient of Variation (GE(2)). These indices differ in their sensitivity to income changes, with the Gini coefficient being more sensitive to income differences around the mode of the distribution, and Generalized Entropy measures increasingly to changes affecting the upper tail as the parameter value in parentheses rises from 0 to 2. We then ask if the observed changes are welfare-improving, and look for Generalized Lorenz dominance that combines information on relative inequality and mean incomes.

The Generalized Entropy measures are the only Lorenz-consistent indices that are additively decomposable by population subgroups. We make use of this property in the next section to see if some groups were more affected than others, and decompose relative inequality within and between age, gender, education, and labour-force and marital status.

The scale-invariance property of the Lorenz-consistent measures implies that inequality remains unchanged as all incomes change by the same proportion: we measure inequality in income shares. This is not the only way forward, and departures from the relative criterion have become increasingly common following the finding in Atkinson and Brandolini (2004) that the evolution of world income inequality was not the same using relative and absolute measures. We thus also calculate absolute measures of inequality. The translation-invariance property of these indices imply now that inequality is unchanged as all incomes change by the same amount: we measure inequality in levels of income. We will here consider the absolute Gini coefficient, the variance of the income distribution, and two versions of the Kolm index with inequality-aversion parameters of $5*10^{-4}$ and $10^{-4}$ (the results are very similar with other parameter values).

Our empirical analysis covers all respondents with valid information on disposable household income. As this latter is measured in bands, we take the mid-point in Euros and in PPP (using 2019 Euros for household final consumption expenditures as the reference). We attribute a value of 12 500 Euros to the open-ended top income category: this value produces the best fit when comparing our relative Gini coefficients in January 2020 to those produced by Eurostat in 2019. Each income figure is then equivalised using the square root of the number of household members, and the resulting value is attributed to each household member. The decomposition by population subgroups is carried out taking as reference the characteristics of the survey respondent.

The analysis sample is made up of the French, German, Italian and Spanish samples. There are 19,237 observations (6,444 individuals) in the analysis sample. Each country represents roughly 25 % of the total sample. We do not include Sweden in the analysis sample as the number of observations is relatively low (fewer than 600 observations in January 2021).

It is natural to compare COME-HERE to the benchmark dataset used in Europe to monitor poverty and inequality, EU-SILC. The latter is a collective enterprise at the European Union level by National Statistical Institutes under the coordination of EUROSTAT with immense expertise in data collection and production. COME-HERE is not on the same scale as EU-SILC, but has the great advantage of already being available and offering multiple observations from 2020 to 2021, which are fundamental qualities for the monitoring of inequality during the pandemic. When we compare our relative Gini coefficients from January 2020 to the latest figures produced from EU-SILC using the same equivalence scale, we find very similar values: the EU-SILC Gini coefficients in 2019 for France, Germany, Spain and Italy were respectively 0.295, 0.293, 0.331 and 0.335; the analogous January 2020 COME-HERE figures were 0.294, 0.302, 0.336 and 0.339. None of these differences are over 0.8 Gini points. We also find that the average equivalised disposable household incomes in COME-HERE in France and Germany in January 2020 are very similar to those that can be calculated from the most recent EU-SILC wave (after applying the same equivalence scale and PPP indices). The picture is somewhat different in Spain and Italy, where the average COME-HERE equivalised disposable household incomes are around 20 % lower than those in EU-SILC, so that we are missing some observations in the right tail of the income distributions. The similarities in terms of Gini coefficients between the two datasets are reassuring for the analysis of inequality. In addition, we are mainly interested in monitoring changes over time, so the level values at baseline are somewhat less of a concern.

Before turning to the analysis of inequality in the next section, we first describe the observed changes in income densities via the summary statistics in Table 1 and the histograms in Fig. 1. In Table 2 we report the evolution of mean equivalised disposable household income in PPP by the respondent's labour-force status in order to help track the economic effects of lockdowns. Average equivalised disposable household income across almost all countries is U-shaped from January 2020 to January 2021. The income fall from January to May 2020 likely reflects the COVID-19 outbreak *per se* and the initial restrictive measures, and the subsequent recovery the governmental compensation schemes implemented throughout 2020 and the reorganisation of economic activities. This U-shaped income pattern is found in all individual countries bar France, where income instead rose fairly steadily from January to

September 2020 before going back to its initial level in January 2021 (although all of the income changes here, over a short period, are necessarily only quite small).

Median income over this twelve-month period is somewhat more stable: this did not change in France and Germany. The U-shaped pattern found for mean income is also apparent in median income in Italy and Spain. Notably, Italy is the only one of our four countries in which neither mean nor median income had recovered to its January 2020 level by January 2021.

In line with Grabka (2021), we report the evolution of mean equivalised disposable household income in PPP by the respondent's labour-force status in Table 2. We here split the sample according to the labour-force status indicated by the respondent among employee, self-employed, unemployed or out of the labour force (OLF), and retired. Italy is the only country where all four labour-force groups experienced income losses during both the early months of the pandemic (from January 2020 to May 2020), and overall (from January 2020 to January 2021). Spain, on the other hand, is the only country where the initial income drops experienced by all groups had recovered by January 2021 (the exception is the small decline of 18 Euros, for the unemployed and OLF). As might be expected from the consequences of lockdowns and restrictions on economic activity, in all the countries the self-employed were the hardest-hit in both the early months of the pandemic and (except in Spain) when we look at the one-year January 2020 to January 2021 change. We confirm the finding in Grabka (2021) that the self-employed in Germany are richer than other labour-force groups, while in our other three countries the differences in mean income between employees, retirees and self-employed are much more nuanced.

The changes in the country income distributions over time are plotted in Fig. 1. The left-hand panel here refers to the January-May 2020 movement, and the right-hand panel to that between January 2020 and January 2021 (the January to September 2020 changes are similar

**Table 1** Equivalised disposable household income in PPP in COME-HERE from January 2020 - Descriptive Statistics

| | Mean | Median | S.D. | Min | Max |
|---|---|---|---|---|---|
| France: | | | | | |
| January 2020 | 2010.3 | 1922.6 | 1140.6 | 283.2 | 11377.5 |
| May 2020 | 2040.7 | 1922.6 | 1174.2 | 262.2 | 8045.1 |
| September 2020 | 2072.4 | 1922.6 | 1180.7 | 283.2 | 11377.5 |
| January 2021 | 2019.1 | 1922.6 | 1127.4 | 262.2 | 11377.5 |
| Germany: | | | | | |
| January 2020 | 2038.6 | 1867.1 | 1189.1 | 275.1 | 11049.5 |
| May 2020 | 2002.9 | 1867.1 | 1149.3 | 238.2 | 11049.5 |
| September 2020 | 2030.6 | 1867.1 | 1148.1 | 301.3 | 11049.5 |
| January 2021 | 2070.4 | 1867.1 | 1173.9 | 238.2 | 11049.5 |
| Italy: | | | | | |
| January 2020 | 1406.9 | 1380.5 | 942.0 | 214.4 | 7458.0 |
| May 2020 | 1294.8 | 1182.4 | 875.2 | 214.4 | 7458.0 |
| September 2020 | 1342.5 | 1260.3 | 825.9 | 214.4 | 7458.0 |
| January 2021 | 1323.6 | 1260.3 | 808.2 | 214.4 | 7458.0 |
| Spain: | | | | | |
| January 2020 | 1337.3 | 1300.1 | 868.7 | 201.9 | 7023.2 |
| May 2020 | 1324.0 | 1186.8 | 873.9 | 228.9 | 7023.2 |
| September 2020 | 1374.6 | 1300.1 | 854.5 | 228.9 | 9932.3 |
| January 2021 | 1386.2 | 1300.1 | 844.9 | 228.9 | 6783 |

*Note*. The figures here refer to the analysis sample from the COME-HERE survey

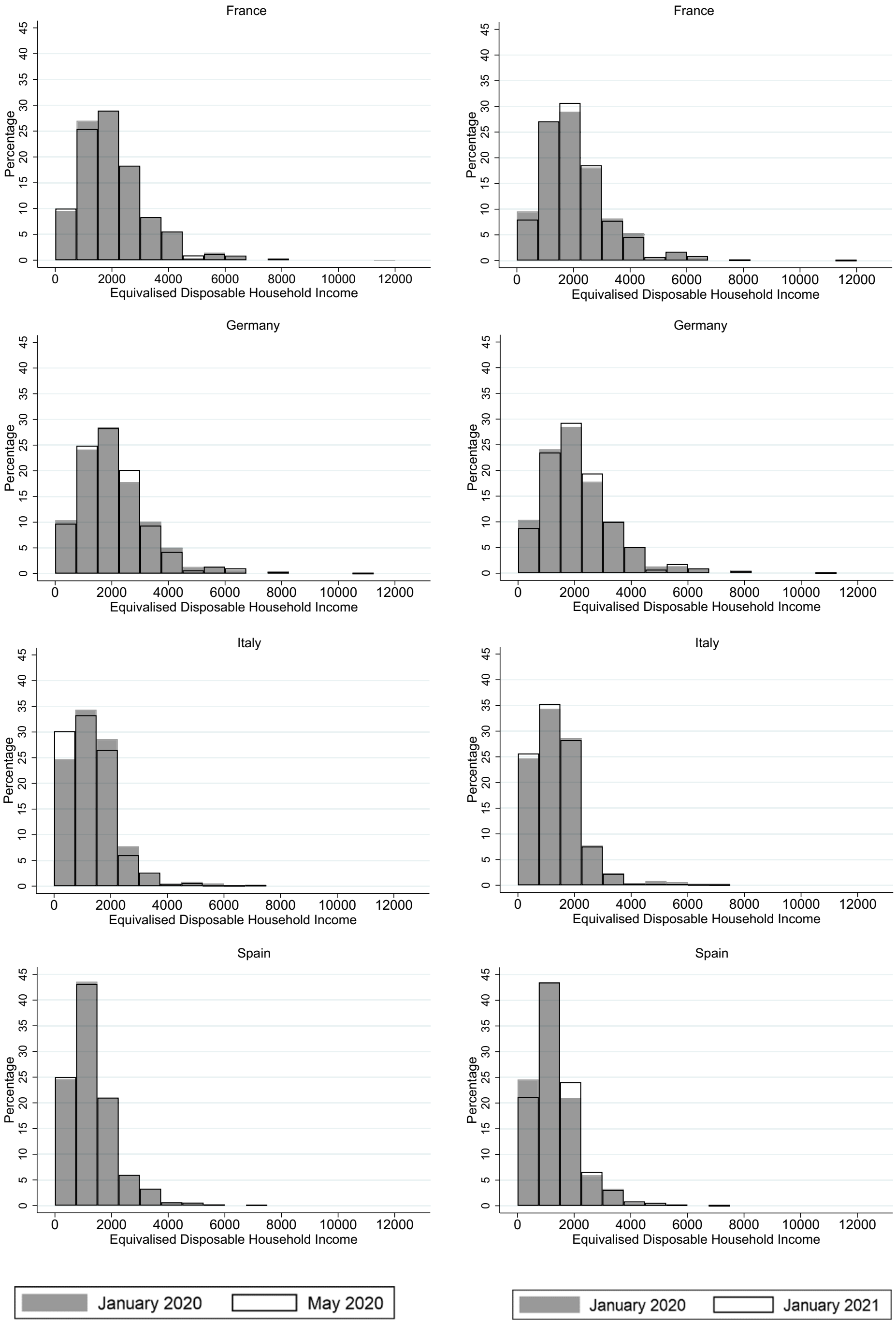

**Fig. 1** The distribution of equivalised disposable household income in COME-HERE from January 2020 to January 2021 in France, Germany, Italy and Spain

**Table 2** Mean equivalised disposable household income in PPP in COME-HERE from January 2020 by Labour-Force Status

| | Employees | Self-employed | Unemployed+OLF | Retired |
|---|---|---|---|---|
| France: | | | | |
| January 2020 | 2039.7 | 2290.5 | 1324.1 | 2206.6 |
| May 2020 | 2095.8 | 2088.5 | 1202.9 | 2219.4 |
| September 2020 | 2109.0 | 2213.8 | 1226.5 | 2241.2 |
| January 2021 | 2035.9 | 1997.8 | 1248.5 | 2208.9 |
| Germany: | | | | |
| January 2020 | 2060.8 | 2811.9 | 1471.2 | 1964.3 |
| May 2020 | 2041.4 | 2487.4 | 1495.4 | 1966.9 |
| September 2020 | 2058.1 | 2393.3 | 1539.5 | 2017.3 |
| January 2021 | 2090.7 | 2627.6 | 1514.7 | 2041.2 |
| Italy: | | | | |
| January 2020 | 1445.5 | 1595.5 | 1033.6 | 1583.0 |
| May 2020 | 1333.0 | 1228.1 | 833.7 | 1550.9 |
| September 2020 | 1360.0 | 1365.5 | 910.3 | 1553.2 |
| January 2021 | 1362.0 | 1385.0 | 846.9 | 1448.4 |
| Spain: | | | | |
| January 2020 | 1358.9 | 1413.0 | 894.5 | 1600.4 |
| May 2020 | 1337.3 | 1236.0 | 870.3 | 1577.8 |
| September 2020 | 1388.7 | 1254.8 | 932.1 | 1576.5 |
| January 2021 | 1387.3 | 1430.2 | 876.4 | 1603.3 |

*Note*. The figures here refer to the analysis sample from the COME-HERE survey to those in the right-hand panel, and are available upon request). It can be seen that the income distribution shifted to the left between January and May in Italy and, to a lesser extent, in Spain. There is a notable higher concentration in the middle-income categories in Germany, while the opposite pattern holds in France where the middle-income categories attract somewhat fewer observations.

Italy is unique with its striking increase in the density in the first bin. Italy was the country that lost the most in the early months of the pandemic: it was the first EU country to be hit hard by COVID-19, and the first to impose a strict lockdown. It is also the EU country with the second-highest share of self-employed among those in work (behind only Greece, according to the most recent Eurostat data published in the online Key European Statistics: this 2019 figure was 21 % in Italy, 15 % in Spain, 11 % in France and 9.5 % in Germany), and we will show below that the self-employed suffered the most of all labour-force groups. Starting in March 2020, the Italian government introduced a number of measures to compensate for the losses due to the pandemic; however, there were complaints about the length of time required to obtain access to government transfers, mainly due to the bank and public-administration procedures. At the same time, wage-compensation schemes for fewer hours of work were extended to cover all employees. Workers outside of the standard wage-guarantee fund again experienced problems in receiving compensation, as their applications also needed to be processed by the region in which they lived. The self-employed received a flat transfer of 600 Euros, later increased to 1000 Euros, regardless of their actual loss. A discussion of the early Italian policy responses to COVID-19 appears in Sanfelici (2020).

Turning to the January 2020-January 2021 distributions on the right of Fig. 1, there is a general shift from the bottom of the distribution towards values in the middle. Italy is again the exception, and is notably the only country where the percentage of respondents with an equivalent income (in PPP) under 750 Euros per month in January 2021 remained higher than in January 2020. In terms of movements throughout the year, the percentage in the bottom income group started to decline in all countries after May 2020, and had already returned to pre-pandemic levels in France and Spain by September 2020. We expect the same recovery to take place very soon in Italy.

We then look at the changes in the histograms by labour-force status (the graphs of these changes corresponding to Fig. 1 are available upon request). As to be expected from the movements in the mean values in Table 2, the numbers that changed the most are those for the self-employed. We thus extend the findings for Germany in Grabka (2021) to the other countries in our sample: in France, Germany and Spain, almost all the losses in income between January and May 2020 were to be found among the self-employed, including the self-employed with the highest incomes. In Italy, all groups lost income over the same period, producing the biggest jump among the four countries of the proportion in the first income bin. Even so, in Italy it was also the self-employed who lost the most in absolute terms between January 2020 and May 2020. The longer-term changes, between January 2020 and January 2021, are more positive than those in the early months of the pandemic, with movements from the bottom towards the adjacent higher income bins, very likely due to targeted policy interventions (the only exception is a very slight increase of Italian retirees in the first two income bins).

## 4 Changing income inequality

We first visually inspect the movements in relative inequality by plotting in Fig. 2 the Lorenz curves for each country between January 2020 and January 2021. Overall, we see a slight shift of the Lorenz curves towards the line of perfect equality in all countries. A formal quantitative measure of this shift is provided by the Gini coefficient (measuring the normalised area between the line of equality and the Lorenz curve) at the foot of the figures. A detailed analysis of the curves reveals Lorenz dominance in German and Spain. There is no Lorenz dominance in France and Italy, as the curves cross. In France the January 2021 curve is below that of January 2020 for higher income shares; in Italy it is below for lower shares.

Figure 3 plots the Gini coefficient and the three Generalized Entropy indices in January 2020, May 2020, September 2020 and January 2021, where January 2020 is normalised to 100 as the baseline (the actual values with 90 % confidence intervals appear in Appendix Table A1). All relative-inequality measures rose between January and May in France, Spain and Italy (significantly so for the latter at the 10 % level). These higher Gini coefficients are in line with the predictions of Almeida et al. (2021) under the scenario of no policy intervention. Although some measures were already in place in May 2020, our results combined with those of Almeida et al. (2021) suggest that the policy responses to the COVID-19 emergency at that time were not immediately effective in tackling the rise in inequality due to differential income losses. The higher Italian Gini coefficient is also very much in line with the simulations in Brunori et al. (2020) comparing the pre-COVID-19 and COVID-19 situations where the only governmental response is lockdown. Germany is an exception here: in May 2020, the Gini coefficient was lower (as indeed were all of the German inequality indices), so that the initial phase of the pandemic was associated with lower inequality.

The German experience at the beginning of the pandemic actually serves as a precursor for the other three countries in our sample as we move to September 2020: relative inequality is lower in September than in January in every country. The difference between most of the

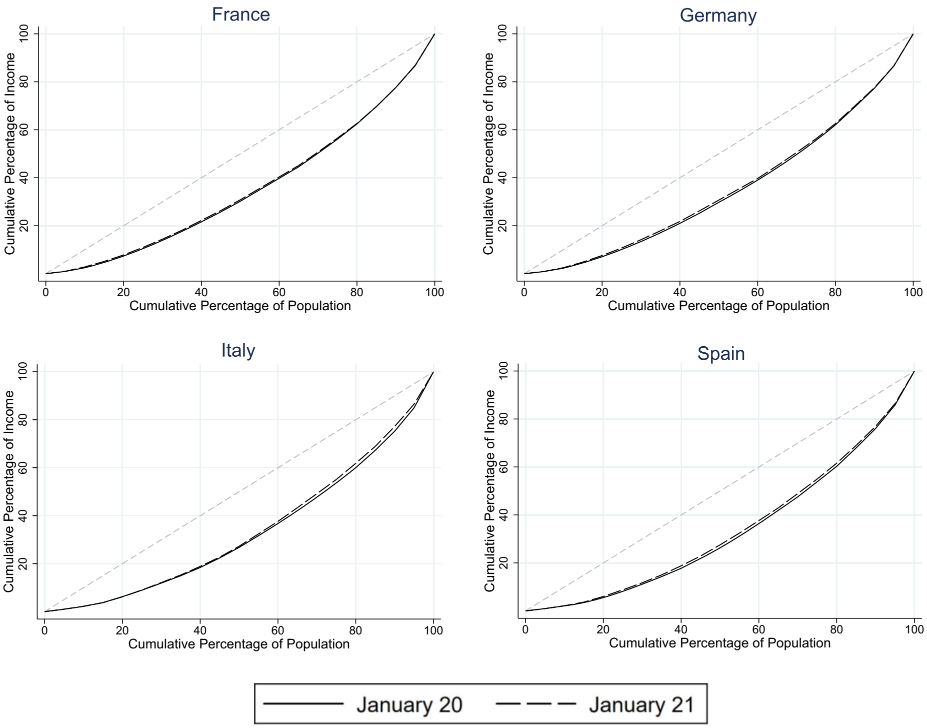

**Fig. 2** Lorenz curves in COME-HERE from January 2020 to January 2021

indices during the two periods are significant in Germany, Italy and Spain at the 10 % level at least. The only exception is the GE(2) measure in France, where we see a slight increase due to greater income differences at the top of the distribution. The largest fall in relative inequality is found for the Italian GE(2) measure. In Italy, the fall in inequality depends on the Generalized Entropy parameter: the larger drop in GE(2) reflects the tightening of the income differences at the top of the distribution. We noted above the opposite shifts in the Lorenz curves in Italy and France, and these are consistent with their different GE(2) experiences. Relative inequality between September 2020 and January 2021 in Germany, Italy and Spain remained stable and at a figure below the January 2020 level. The measures of relative inequality in France continued to fall and, as for the rest of the analysis sample, were all below their initial level.

The overall picture of the distribution of income in Europe during the pandemic can then be split into two periods. The advent of COVID-19 increased relative income inequality in the first period (except in Germany); however, in the second period the evolution of the pandemic and the effect of various policy interventions has more than reversed this initial widening of inequality.

Are these longer-term changes welfare improving? Following Shorrocks (1983), we can combine relative inequality changes from the Lorenz curves with the evolution of mean income and look for Generalized Lorenz (GL) domination in Fig. 4. The GL dominant distribution is preferred by all increasing, S-concave social welfare functions. Overall, we observe an upward shift of the GL curves in France, Germany and Spain. There is strict dominance of the 2021 GL curve over that from 2020 in France and Spain, implying that the

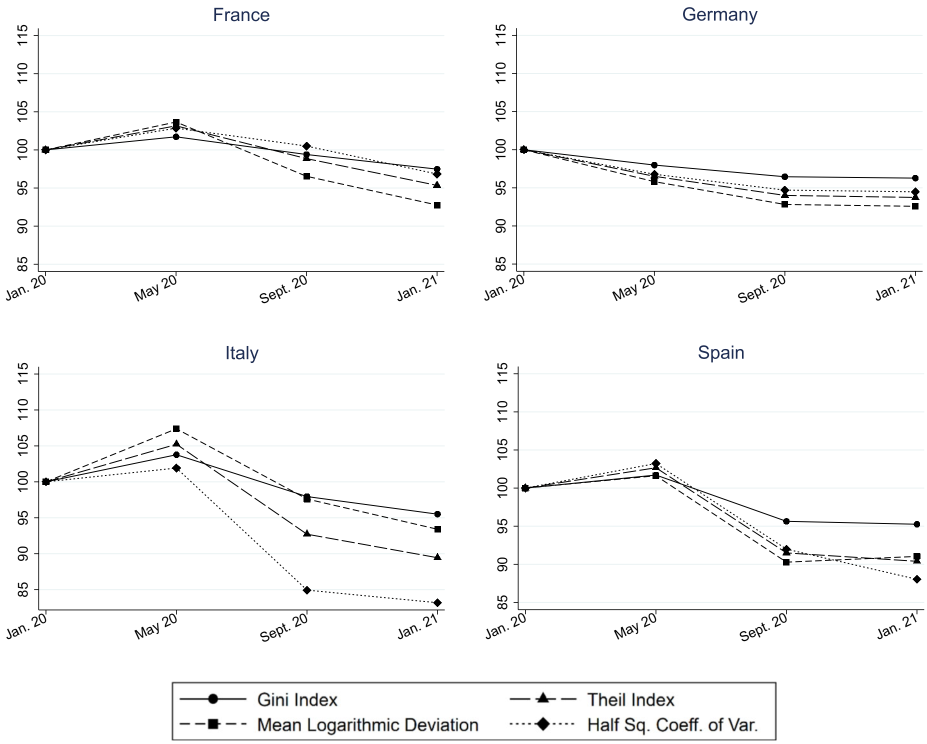

**Fig. 3** The evolution of relative income inequality in COME-HERE from January 2020 to January 2021

income distributions of January 2021 are unambiguously associated with higher social welfare in these two countries. This also seems to be roughly the case in Germany, although the 2021 GL curve is below the 2020 curve twice at the beginning of the curves (for values of $p$ equal to 0.005 and 0.011). Italy stands out, with the income distribution changes being clearly welfare-decreasing: the 2021 GL curve is always below the 2020 curve for $p$ greater than 0.05, and overlapping before this value.

Tables 3, 4 and 5 contain the decompositions of the three Generalized Entropy measures in January 2020 and January 2021 by five individual characteristics: gender, age (above/below 50), education (post-secondary education vs. otherwise) being in a couple, and labour-force status (employee, self-employed, unemployed and OLF, and retired). First, in all countries and periods the within components explain by far the largest part of total inequality (both in levels and changes). This result is commonplace in the inequality literature.

We find that inequality within groups fell over time. The results for the between group components are mixed, and for 13 out of the 60 country values there is no change. One of the largest changes in the between component over time is with respect to education in Spain. There is a drop in inequality between the weighted income means by education in Italy and Spain but this figure rises in France and Germany. We also find an increase in the between partnered vs. non-partnered component in France and Germany. This result is unsurprising if we consider partnership to provide insurance in times of uncertainty. In France, Spain and Germany, the between component among labour-force status groups is reduced between January 2020 and January 2021. This is consistent with the figures of Table 2 and the

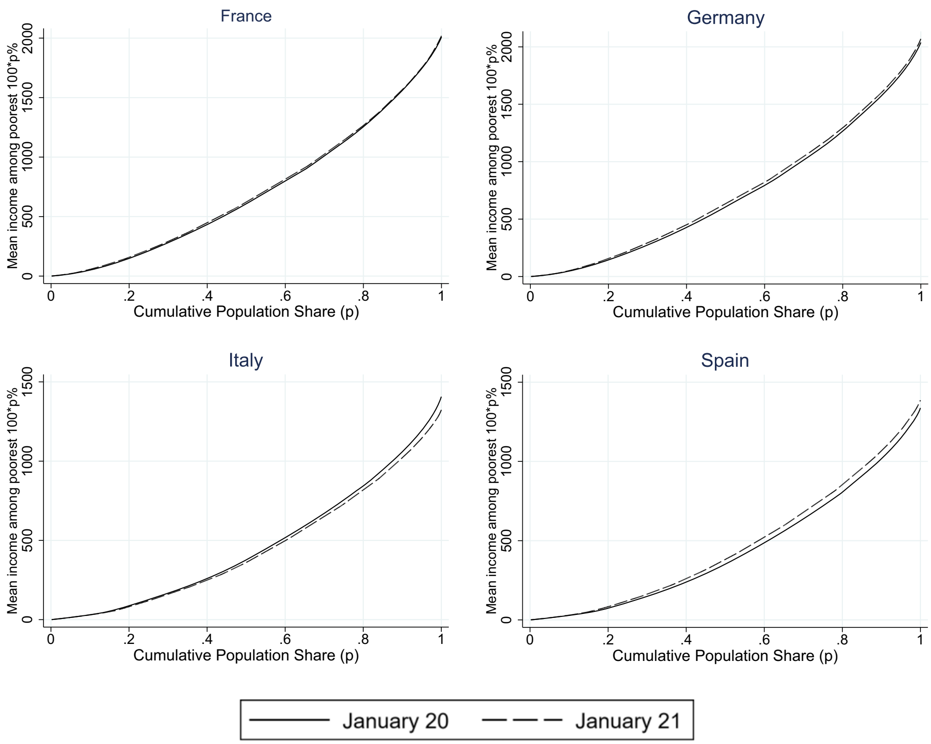

**Fig. 4** Generalised Lorenz curves in COME-HERE from January 2020 to January 2021

conclusions of Grabka (2021): those who lost the most during the pandemic are the self-employed who were, in January 2020, the group with the highest levels of income on average.

The literature we surveyed in Section 2 above concluded that the COVID-19 pandemic had hit women harder than men on the labour market. We might then expect to find greater income inequality between men and women. However, we here measure inequality in equivalised disposable household income. As such, government transfers and income pooling at the household level may counterbalance the unequal gender consequences of COVID-19 on the labour market. There is no increase in inequality between men and women in any of the countries we examine here: the between component of inequality for gender is unchanged at the three-digit level in all countries bar Germany. As such, transfers and income pooling between household members have helped offset the pandemic's gendered labour-market consequences.

We last turn to absolute inequality. Appendix Table A2 lists the index values in January 2020, May 2020, September 2020, and January 2021 (with their 90 % confidence intervals), which are plotted in Fig. 5 using January as the baseline. Two different patterns emerge across countries. While relative inequality increased everywhere between January and May except in Germany, absolute inequality rose only in France and, to a lesser extent, Spain. By January 2021, absolute inequality was below its January 2020 value everywhere. Although we do not observe the exact changes in household income, this drop in absolute inequality may well reflect that the poorest households benefited more from government support during the pandemic. Overall, absolute differences in equivalent household incomes are narrower because on average those at the bottom lost less than the rest in absolute terms.

**Table 3** Mean logarithmic deviation index (GE(0)) – Decomposition of income inequality

| | January 2020 | | | January 2021 | | |
|---|---|---|---|---|---|---|
| France | Total | Within | Between | Total | Within | Between |
| Gender | 0.161 | 0.155 | 0.006 | 0.149 | 0.143 | 0.006 |
| Age | 0.161 | 0.157 | 0.004 | 0.149 | 0.147 | 0.002 |
| Education | 0.161 | 0.152 | 0.009 | 0.149 | 0.139 | 0.010 |
| Partnered | 0.161 | 0.152 | 0.009 | 0.149 | 0.139 | 0.010 |
| Labour Force Status | 0.161 | 0.152 | 0.009 | 0.149 | 0.141 | 0.006 |
| Germany | Total | Within | Between | Total | Within | Between |
| Gender | 0.172 | 0.169 | 0.003 | 0.159 | 0.154 | 0.005 |
| Age | 0.172 | 0.172 | 0.000 | 0.159 | 0.159 | 0.000 |
| Education | 0.172 | 0.160 | 0.012 | 0.159 | 0.145 | 0.014 |
| Partnered | 0.172 | 0.168 | 0.004 | 0.159 | 0.154 | 0.005 |
| Labour Force Status | 0.172 | 0.164 | 0.008 | 0.159 | 0.154 | 0.005 |
| Italy | Total | Within | Between | Total | Within | Between |
| Gender | 0.207 | 0.205 | 0.002 | 0.193 | 0.191 | 0.003 |
| Age | 0.207 | 0.207 | 0.001 | 0.193 | 0.193 | 0.000 |
| Education | 0.207 | 0.197 | 0.010 | 0.193 | 0.184 | 0.009 |
| Partnered | 0.207 | 0.202 | 0.005 | 0.193 | 0.189 | 0.005 |
| Labour Force Status | 0.207 | 0.197 | 0.010 | 0.193 | 0.183 | 0.010 |
| Spain | Total | Within | Between | Total | Within | Between |
| Gender | 0.216 | 0.210 | 0.006 | 0.197 | 0.191 | 0.005 |
| Age | 0.216 | 0.208 | 0.008 | 0.197 | 0.192 | 0.005 |
| Education | 0.216 | 0.203 | 0.013 | 0.197 | 0.187 | 0.010 |
| Partnered | 0.216 | 0.211 | 0.005 | 0.197 | 0.195 | 0.002 |
| Labour Force Status | 0.216 | 0.203 | 0.013 | 0.197 | 0.186 | 0.011 |

*Notes*. These figures refer to the analysis sample from the COME-HERE survey. “Income” refers to equivalised disposable household monthly income in Euros and PPP

## 5 Robustness checks

We here present a number of robustness tests to evaluate the stability and reliability of our inequality trends to first sample selection and then income being reported in intervals.

Only 64 % of the COME-HERE respondents who reported their household income in January 2020 also provided a figure for January 2021. As attrition in COME-HERE is non-random (attrition falls with age and education, although it does not depend on the level of income), Figs. 3 and 4 may confound the evolution of relative and absolute inequality due to the COVID-19 pandemic with changes in the sample composition. We examine this possibility in two ways. We first carry out our analysis on the unbalanced panel using sample weights that guarantee national representativeness in terms of age, gender and region of residence (as was the case by stratification in the first wave). The results of these exercises appear in Figures A1 and A2. The changes in relative and absolute inequality depicted there are similar to those in Figs. 3 and 4, except for France where GE(1) and GE(2) are not below their initial level in January 2021. Changes in sample composition do not then seem to lie behind our conclusions regarding the overall evolution of inequality. We also replicate our analysis using an Inverse Probability Weighting procedure to account for non-random attrition: the resulting profiles in Figures A3 and A4 are similar to those in Figures A1 and A2.

The COME-HERE survey has the structure of what is usually called ‘grouped-data’, as household disposable income is measured in bands. As noted above, we use the mid-points of the income bands to calculate equivalised household disposable income. As such, we do not take into account within-income-band inequality. Although Von Hippel et al. (2017) argue that

**Table 4** Theil index (GE(1)) – Decomposition of income inequality

| | January 2020 | | | January 2021 | | |
|---|---|---|---|---|---|---|
| France | Total | Within | Between | Total | Within | Between |
| Gender | 0.147 | 0.141 | 0.006 | 0.140 | 0.134 | 0.006 |
| Age | 0.147 | 0.143 | 0.004 | 0.140 | 0.138 | 0.002 |
| Education | 0.147 | 0.138 | 0.009 | 0.140 | 0.130 | 0.010 |
| Partnered | 0.147 | 0.139 | 0.008 | 0.140 | 0.131 | 0.009 |
| Labour Force Status | 0.147 | 0.139 | 0.008 | 0.140 | 0.133 | 0.007 |
| Germany | Total | Within | Between | Total | Within | Between |
| Gender | 0.155 | 0.152 | 0.003 | 0.145 | 0.141 | 0.005 |
| Age | 0.155 | 0.155 | 0.000 | 0.145 | 0.145 | 0.000 |
| Education | 0.155 | 0.142 | 0.012 | 0.145 | 0.131 | 0.015 |
| Partnered | 0.155 | 0.151 | 0.004 | 0.145 | 0.140 | 0.005 |
| Labour Force Status | 0.155 | 0.147 | 0.008 | 0.145 | 0.140 | 0.005 |
| Italy | Total | Within | Between | Total | Within | Between |
| Gender | 0.193 | 0.191 | 0.002 | 0.172 | 0.170 | 0.003 |
| Age | 0.193 | 0.192 | 0.001 | 0.172 | 0.172 | 0.000 |
| Education | 0.193 | 0.183 | 0.010 | 0.172 | 0.163 | 0.009 |
| Partnered | 0.193 | 0.188 | 0.005 | 0.172 | 0.168 | 0.005 |
| Labour Force Status | 0.193 | 0.184 | 0.009 | 0.172 | 0.163 | 0.009 |
| Spain | Total | Within | Between | Total | Within | Between |
| Gender | 0.192 | 0.186 | 0.006 | 0.174 | 0.168 | 0.005 |
| Age | 0.192 | 0.185 | 0.008 | 0.174 | 0.169 | 0.005 |
| Education | 0.192 | 0.179 | 0.013 | 0.174 | 0.164 | 0.010 |
| Partnered | 0.192 | 0.188 | 0.005 | 0.174 | 0.172 | 0.002 |
| Labour Force Status | 0.192 | 0.180 | 0.012 | 0.174 | 0.164 | 0.010 |

*Notes*. These figures refer to the analysis sample from the COME-HERE survey. "Income" refers to equivalised disposable household monthly income in Euros and PPP

using mid-points in the case of grouped-data is the best approach for the estimation of inequality indices when the true income-distribution parameters are unknown, we here appeal to the 'split-histogram' technique (Cowell and Metha 1982) to re-estimate our main results. As expected, the inequality measures are about 2–5 % larger with this technique (the time series are available upon request). The resulting trends are plotted in Figures A5 and A6, and are not different from those in the baseline results: there is decreasing relative and absolute inequality, except in France where the results are more contrasted.

An additional concern with grouped data is that we only observe income changes when respondents switch from one of our seven income bands to another across survey waves. This means that we do not measure income shocks (either positive or negative) for respondents who remain in the same income band from one survey wave to the next. This problem is particularly salient in the top and the bottom income bands, with the importance in distributive studies of the poorest and richest individuals. Fortunately, there are additional survey income questions that help us to address potential changes *within* income bands from January 2020 to May 2020, from May to September 2020, and from September 2020 to November 2020 (no survey was fielded in January 2021).

COME-HERE respondents in Wave 1, around May 1st 2020, were asked to report whether their income had changed between January 2020 and the date of interview; an analogous question in Wave 3, around September 5th 2020, referred to income changes between May 2020 and the interview date. In Wave 4, around November 19th 2020 the question referred to income changes between September 2020 and the interview date. If their income had changed over these periods, respondents then expressed their current income as a

**Table 5** Half the square of the coefficient of variation (GE(2)) – Decomposition of income inequality

| | January 2020 | | | January 2021 | | |
|---|---|---|---|---|---|---|
| France | Total | Within | Between | Total | Within | Between |
| Gender | 0.161 | 0.155 | 0.006 | 0.156 | 0.149 | 0.006 |
| Age | 0.161 | 0.157 | 0.004 | 0.156 | 0.153 | 0.002 |
| Education | 0.161 | 0.152 | 0.009 | 0.156 | 0.146 | 0.010 |
| Partnered | 0.161 | 0.153 | 0.008 | 0.156 | 0.147 | 0.009 |
| Labour Force Status | 0.161 | 0.153 | 0.007 | 0.156 | 0.149 | 0.006 |
| Germany | Total | Within | Between | Total | Within | Between |
| Gender | 0.170 | 0.167 | 0.003 | 0.161 | 0.156 | 0.005 |
| Age | 0.170 | 0.170 | 0.000 | 0.161 | 0.160 | 0.000 |
| Education | 0.170 | 0.157 | 0.013 | 0.161 | 0.146 | 0.015 |
| Partnered | 0.170 | 0.166 | 0.004 | 0.161 | 0.156 | 0.005 |
| Labour Force Status | 0.170 | 0.162 | 0.008 | 0.161 | 0.156 | 0.005 |
| Italy | Total | Within | Between | Total | Within | Between |
| Gender | 0.224 | 0.222 | 0.002 | 0.186 | 0.184 | 0.003 |
| Age | 0.224 | 0.224 | 0.000 | 0.186 | 0.186 | 0.000 |
| Education | 0.224 | 0.214 | 0.010 | 0.186 | 0.177 | 0.009 |
| Partnered | 0.224 | 0.219 | 0.005 | 0.186 | 0.182 | 0.005 |
| Labour Force Status | 0.224 | 0.216 | 0.008 | 0.186 | 0.178 | 0.008 |
| Spain | Total | Within | Between | Total | Within | Between |
| Gender | 0.211 | 0.205 | 0.006 | 0.186 | 0.180 | 0.005 |
| Age | 0.211 | 0.203 | 0.008 | 0.186 | 0.181 | 0.005 |
| Education | 0.211 | 0.198 | 0.013 | 0.186 | 0.176 | 0.010 |
| Partnered | 0.211 | 0.206 | 0.005 | 0.186 | 0.184 | 0.002 |
| Labour Force Status | 0.211 | 0.199 | 0.011 | 0.186 | 0.177 | 0.009 |

*Notes*. These figures refer to the analysis sample from the COME-HERE survey. “Income” refers to equivalised disposable household monthly income in Euros and PPP

percentage of their initial income (i.e. in January, May or November) using the following intervals: *“0 %”, “1–24 %”, “25–49 %”, “50–74 %”, “75–99 %”*, and *“>100 %”*.

75 % of COME-HERE respondents report being in the same one of our seven income bands from one survey wave to the next. We wish to know whether their income had changed within this band. Of this 75 %, almost three-quarters reported no income change in the above questions. Amongst the 25 % who did report an income change (while remaining in the same income band between the two survey waves), the vast majority replied *“75–99 %”* to the income-ratio question. As such, the largest possible income change within bands that could have occurred would be a fall of 25 % for one quarter of 75 % of the sample.

We can evaluate the impact of these relatively few and small changes in household disposable income within income bands between two consecutive waves by multiplying the mid-points of the income bands in question by the mid-points of the income-change categories. We consider the income-ratio category “*>100 %*” to correspond to an income rise of 20 % (as under 4 % of respondents report this change, this 20 % figure has almost no effect on the results).

We then recalculate the change in inequality over time, including both individuals who change income bands (25 % of the sample), those who report that their income has changed over time while remaining within the same band (one quarter of the other 75 %), and those within the same band with no reported income change (the remaining three quarters of the 75 %).

The results appear in Figures A7 and A8. The trends in relative and absolute inequality when accounting for income changes within the same income band turn out to be very similar

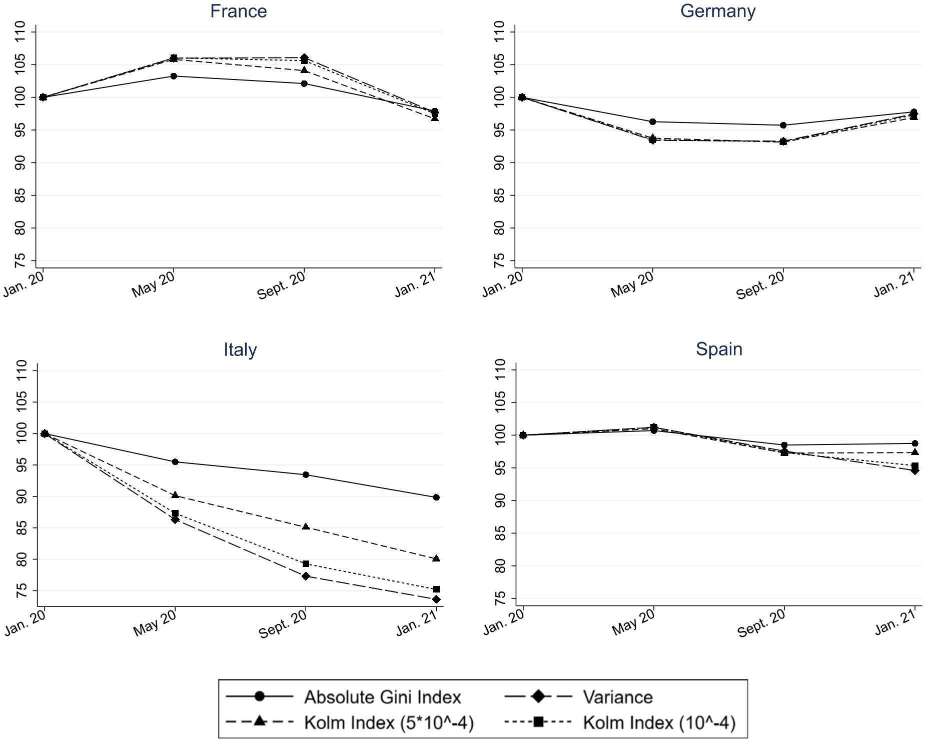

**Fig. 5** The evolution of absolute income inequality in COME-HERE from January 2020 to January 2021

to those in the baseline. The categorical income information in COME-HERE does not unduly influence our inequality conclusions.

## 6 Conclusions

Longitudinal data from the COME-HERE survey covering France, Germany, Italy and Spain reveals a fall in relative inequality between January 2020 and January 2021. The evolution of relative inequality over 2020 was not monotonic: inequality mostly increased from January to May 2020 before dropping back below its pre-COVID level in September 2020. The fall in relative inequality in France was slower than in the other countries. The absolute inequality in equivalised disposable household income also fell in all four countries. We show that due to the pandemic some households lost more than others, with the self-employed being hit the hardest, confirming the recent findings in Grabka (2021) for Germany using SOEP data. The income-support policies to address losses due to COVID-19 have focussed more on those towards the bottom of the income distribution rather than compensating all groups equally.

Although this paper is one of the first to track the changes in relative and absolute inequality across different European countries via a harmonised survey, it is not without limitations. We do, however, believe that these latter can be addressed in future research. First, we have seen some differences in patterns using data from only four countries: surveys including more (or at least different) countries should be explored to improve our understanding of the effects of COVID-19 on both national and international inequality. Second, the question of the mechanisms remains open, and we would like to better understand the efficiency of the various policy responses. Last, the latest survey wave that we analysed here referred to disposable household income in January 2021, in the middle of the third wave of COVID-19. More-recent data would allow us to see whether the compensation schemes in place were sufficient to avoid a potential new jump in inequality during the restrictions imposed in 2021. Addressing these questions and limitations constitutes a promising and necessary field of investigation for future research.

**Supplementary Information** The online version contains supplementary material available at https://doi.org/10.1007/s10888-021-09499-2.

**Open Access** This article is licensed under a Creative Commons Attribution 4.0 International License, which permits use, sharing, adaptation, distribution and reproduction in any medium or format, as long as you give appropriate credit to the original author(s) and the source, provide a link to the Creative Commons licence, and indicate if changes were made. The images or other third party material in this article are included in the article's Creative Commons licence, unless indicated otherwise in a credit line to the material. If material is not included in the article's Creative Commons licence and your intended use is not permitted by statutory regulation or exceeds the permitted use, you will need to obtain permission directly from the copyright holder. To view a copy of this licence, visit http://creativecommons.org/licenses/by/4.0/.

## Supplementary Information

ESM 1 (DOCX 1086 kb)
