# Supplementary material for: The fall in income inequality during COVID-19 in four European countries
Source: J Econ Inequal. 2021 Aug 8;19(3):489–507. doi: 10.1007/s10888-021-09499-2 (PMC8349307; doi:10.1007/s10888-021-09499-2)
Supplement: Supplementary file 1 — (DOCX 1086 kb) [file 10888_2021_9499_MOESM1_ESM.docx]

**Appendix:**

This Appendix describes the time series for the inequality indices (Tables A1 and A2). Table A1 shows that all relative-inequality measures, except in Germany, rose between January and May 2020 (and significantly so at the 10% level at least in Italy). However, relative inequality was lower in September 2020 than it was in January 2020 in every country, with these differences being significant at the 10% level in Germany and Spain. The difference between the Gini indices in Spain in January and September is statistically significant at the 5% level. This is unsurprising: Spain is the only country where the cumulative shares of income were significantly higher at conventional levels for 8 out the 10 deciles of the income distribution between January and September (results available upon request). We find similar hump-shaped profiles in the other measures of relative inequality. As for the Gini index, most of the General Entropy measures are significantly lower in September 2020 than in January 2020 in every country bar France. Our conclusions remain somewhat the same in January 2021.

We then turn to absolute inequality in Table A2. Between January and May 2020, absolute inequality rose only in France and, to a lesser extent, Spain. By January 2021, absolute inequality was below its January 2020 value everywhere (and significantly so for Italy).

Figures A1 to A8 depict the evolution of all the afore-mentioned indices between January and September 2020 when we take into account the issues of national representativeness, attrition, grouped-data and unobserved income changes within income bands. They reveal that none of these issues has a substantial effect on our conclusions about the evolution of relative and absolute inequality over the course of 2020.

Table A1: Relative Income Inequality Indices in COME-HERE from January 2020 to January 2021

|  | 2020 | | |  | 2021 |
| --- | --- | --- | --- | --- | --- |
| ***Gini Coefficient*** | January | May | September |  | January |
| France | 0.294  [0.283;0.305] | 0.299  [0.286;0.311] | 0.293  [0.282;0.303] |  | 0.287  [0.274;0.299] |
| Germany | 0.302  [0.292;0.312] | 0.296  [0.284;0.308] | 0.290  [0.278;0.302] |  | 0.291  [0.279;0.303] |
| Italy | 0.336  [0.324;0.348] | 0.349  [0.336;0.362] | 0.328  [0.315;0.341] |  | 0.321  [0.307;0.335] |
| Spain | 0.339  [0.328;0.350] | 0.345  [0.331;0.358] | 0.323  [0.310;0.336] |  | 0.323  [0.309;0.337] |
| ***Theil Index*** | January | May | September |  | January |
| France | 0.147  [0.137;0.157] | 0.152  [0.139;0.164] | 0.146  [0.135;0.156] |  | 0.140  [0.128;0.152] |
| Germany | 0.155  [0.145;0.165] | 0.150  [0.138;0.162] | 0.145  [0.132;0.157] |  | 0.145  [0.133;0.158] |
| Italy | 0.193  [0.179;0.207] | 0.203  [0.188;0.218] | 0.178  [0.163;0.192] |  | 0.172  [0.158;0.187] |
| Spain | 0.192  [0.180;0.205] | 0.198  [0.181;0.213] | 0.175  [0.160;0.190] |  | 0.174  [0.160;0.188] |
| ***Mean Logarithmic Deviation*** | January | May | September |  | January |
| France | 0.161  [0.149;0.173] | 0.167  [0.153;0.181] | 0.156  [0.145;0.167] |  | 0.149  [0.136;0.163] |
| Germany | 0.172  [0.161;0.183] | 0.165  [0.150;0.179] | 0.158  [0.144;0.172] |  | 0.159  [0.144;0.174] |
| Italy | 0.207  [0.193;0.221] | 0.222  [0.207;0.238] | 0.201  [0.185;0.217] |  | 0.193  [0.178;0.209] |
| Spain | 0.216  [0.203;0.230] | 0.220  [0.204;0.234] | 0.194  [0.179;0.210] |  | 0.197  [0.181;0.213] |
| ***Half the Square of Coefficient of Variation*** | January | May | September |  | January |
| France | 0.161  [0.148;0.174] | 0.166  [0.150;0.181] | 0.162  [0.149;0.176] |  | 0.156  [0.140;0.171] |
| Germany | 0.170  [0.157;0.183] | 0.165  [0.149;0.180] | 0.160  [0.144;0.176] |  | 0.161  [0.144;0.177] |
| Italy | 0.224  [0.202;0.246] | 0.228  [0.205;0.251] | 0.189  [0.170;0.208] |  | 0.186  [0.166;0.207] |
| Spain | 0.211  [0.193;0.228] | 0.218  [0.195;0.240] | 0.193  [0.170;0.216] |  | 0.186  [0.167;0.204] |

*Notes*. These figures refer to the analysis sample from the COME-HERE survey. The figures in square brackets show the 90% confidence intervals.

Table A2: Absolute Income Inequality Indices in COME-HERE from January 2020 to January 2021

|  | 2020 | | |  | 2021 |
| --- | --- | --- | --- | --- | --- |
| ***Absolute Gini Coefficient*** | January | May | September |  | January |
| France | 591.1  [564,5;617.8] | 610.4  [579.9;640.9] | 606.6  [576.8;636.5] |  | 578.8  [545.6;612.0] |
| Germany | 615.7  [589.0;642.5] | 592.8  [562.5;623.1] | 589.6  [557.8;621.4] |  | 602.0  [563.9;640.2] |
| Italy | 472.9  [448.7;497.1] | 451.6  [426.9;476.4] | 440.4  [420.3;460.6] |  | 424.9  [404.2;445.6] |
| Spain | 453.5  [435.1;471.9] | 456.7  [433.8;479.6] | 444.2  [422.6;465.9] |  | 447.8  [425.7;468.9] |
| ***Variance*** | January | May | September |  | January |
| France | 1300857  [1172139;1430666] | 1378857  [1230393;1528938] | 1394062  [1249308;1540479] |  | 1270925  [1108494;1434968] |
| Germany | 1413852  [1283226;1545671] | 1320901  [1171891;1471417] | 1318019  [1161298;1476384] |  | 1377944  [1191511;1566383] |
| Italy | 887270  [776415;998964] | 765908  [668582;864216] | 682161  [604702;760495] |  | 653235  [575759;731638] |
| Spain | 754720  [680171;829931] | 763753  [668642;859789] | 730172  [631173;830060] |  | 713912  [623511;805252] |
| ***Kolm Index (5*10^-4^)*** | January | May | September |  | January |
| France | 212.2  [198.3;226 .1] | 224.5  [209.8;239.2] | 222.7  [208.2;237.2] |  | 205.2  [190.7;219.7] |
| Germany | 240.6  [225.0;256.2] | 225.5  [207.6;243.4] | 224.1  [204.2;244.0] |  | 233.1  [213.1;253.1] |
| Italy | 163.2  [151.6;174.8] | 147.1  [135.5;158.7] | 138.1  [129.2;147.0] |  | 130.7  [121.8;139.6] |
| Spain | 164.8  [154.4;175.2] | 166.4  [154.1;178.7] | 158.9  [145.8;178.7] |  | 160.4  [147.3;173.5] |
| ***Kolm Index (10^-4^)*** | January | May | September |  | January |
| France | 50.14  [45.96;54.32] | 53.16  [48.33;57 .99] | 53.48  [48.63;58.33] |  | 48.88  [45.03;53.73] |
| Germany | 57.50  [52.47;62.53] | 53.77  [48.23;59.31] | 53.56  [47.47;59.65] |  | 55.95  [49.86;62.04] |
| Italy | 39.47  [36.00;42.94] | 34.46  [31.05;37 .87] | 31.11  [28.67;33.55] |  | 29.68  [27.24;32.12] |
| Spain | 38.37  [35.29;41.44] | 38.83  [35.31;42.35] | 37.01  [32.90;41.12] |  | 36.58  [32.47;40.69] |

*Notes*. These figures refer to the analysis sample from the COME-HERE survey. The figures in square brackets show the 90% confidence intervals.

Figure A1: The evolution of Relative Income Inequality in COME-HERE from January 2020 to January 2021 – Cross-sectional Sample Weights


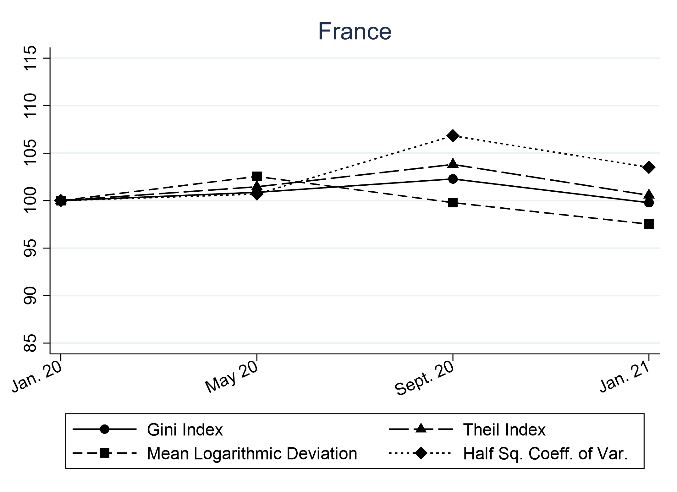

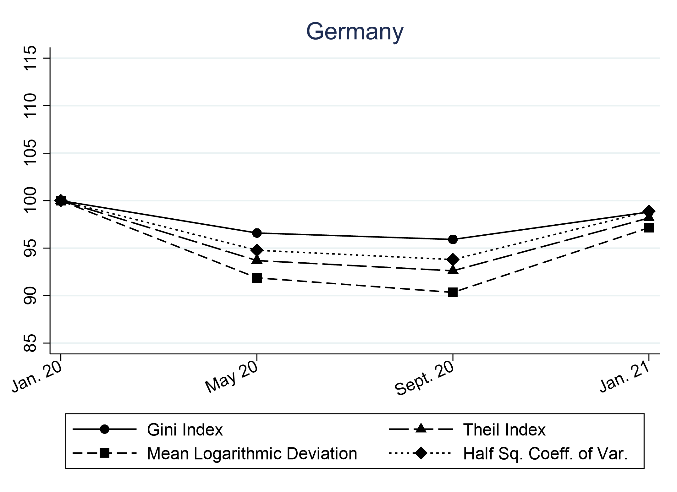

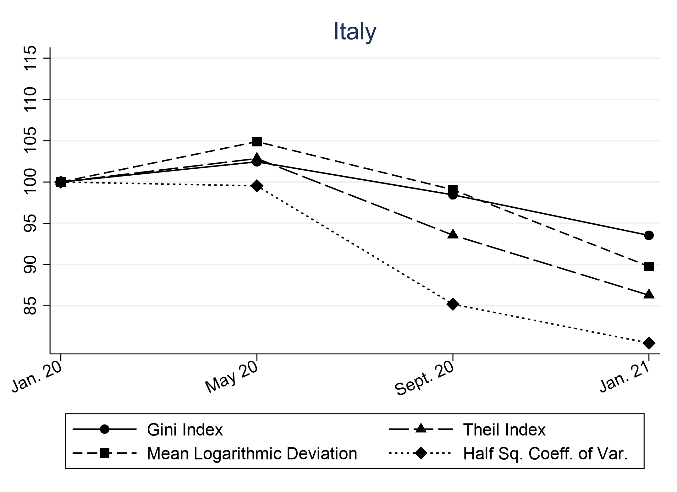

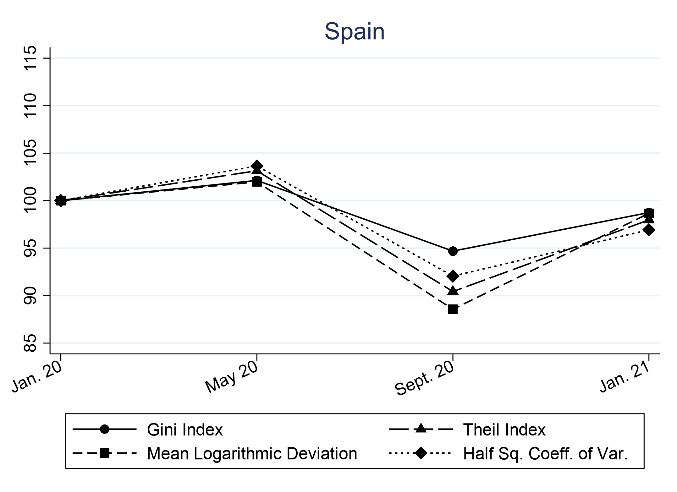


*Notes*. These figures refer to the analysis sample from the COME-HERE survey. “Income” refers to equivalised disposable household monthly income in Euros and PPP.

Figure A2: The evolution of Absolute Income Inequality in COME-HERE from January 2020 to January 2021 – Cross-sectional Sample Weights


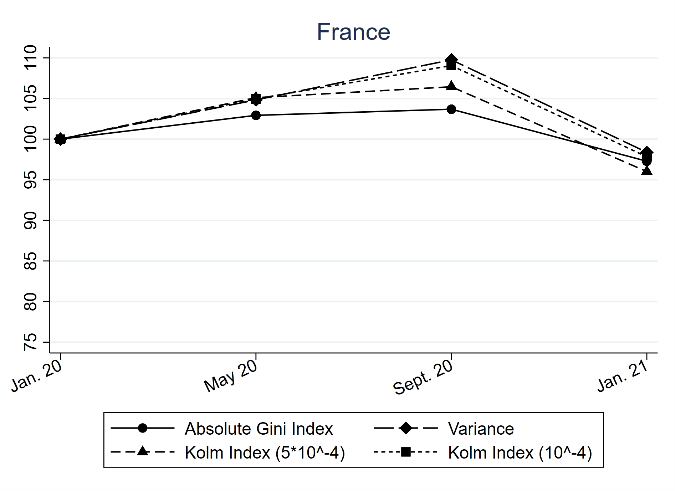

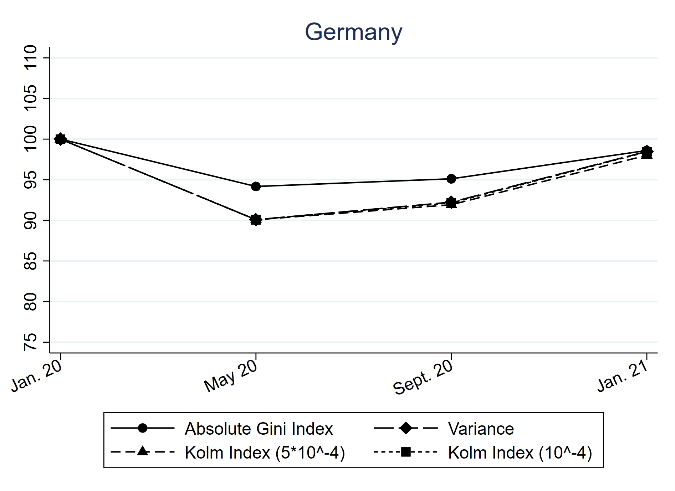

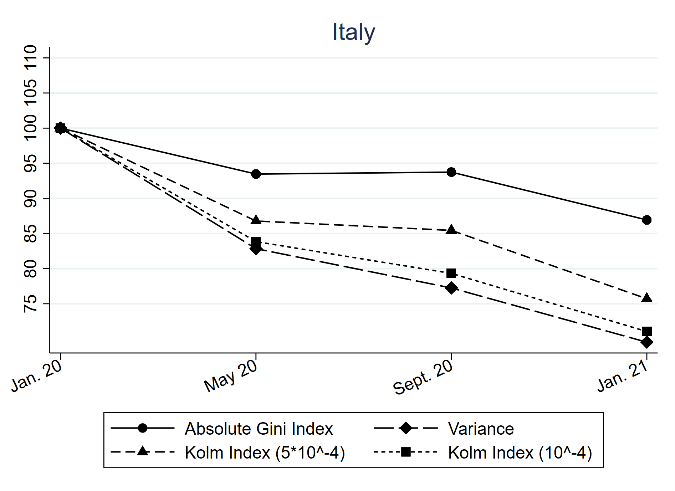

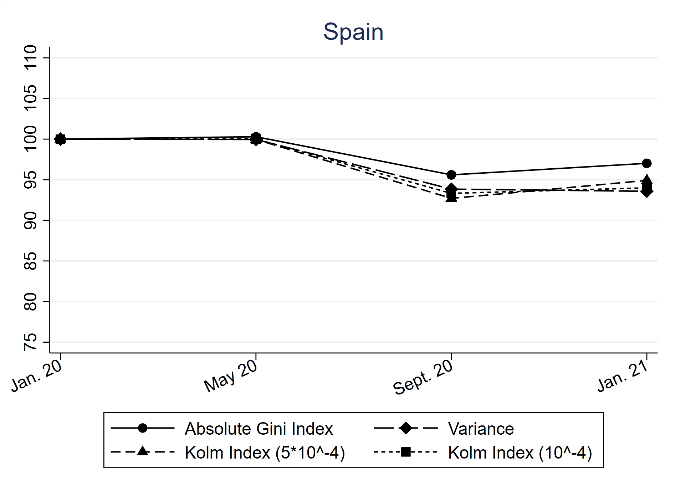


*Notes*. These figures refer to the analysis sample from the COME-HERE survey. “Income” refers to equivalised disposable household monthly income in Euros and PPP.

Figure A3: The evolution of Relative Income Inequality in COME-HERE from January 2020 to January 2021 - Longitudinal Sample Weights


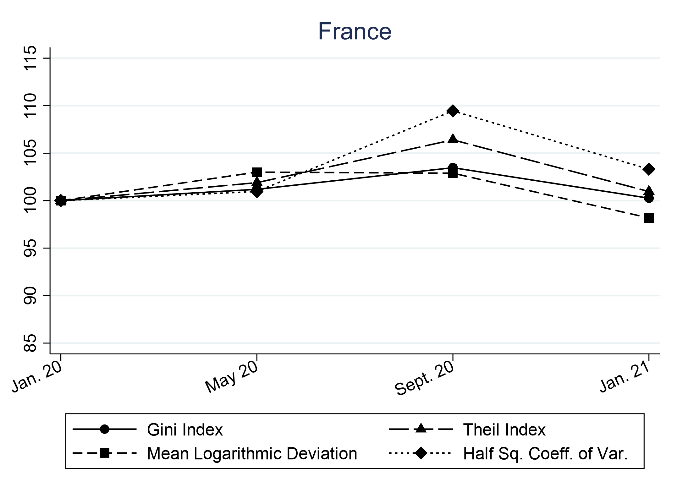

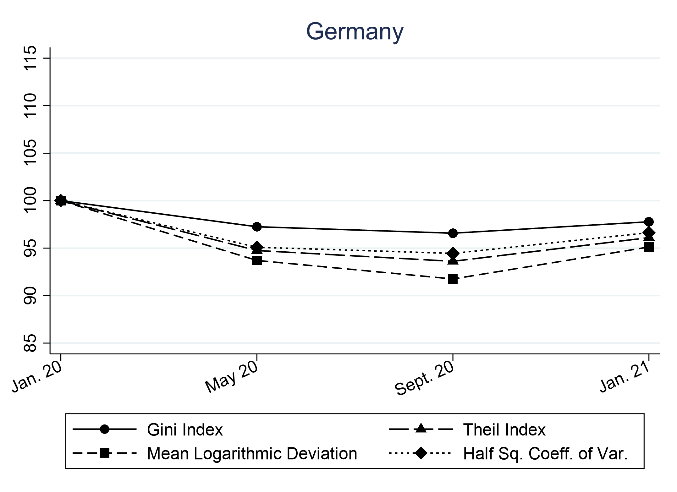

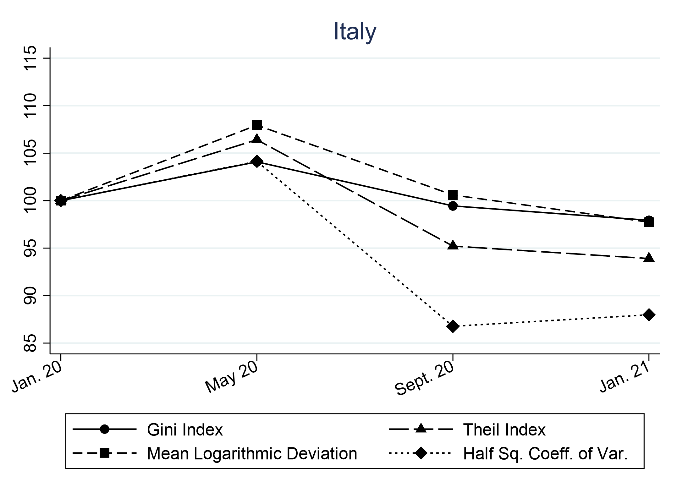

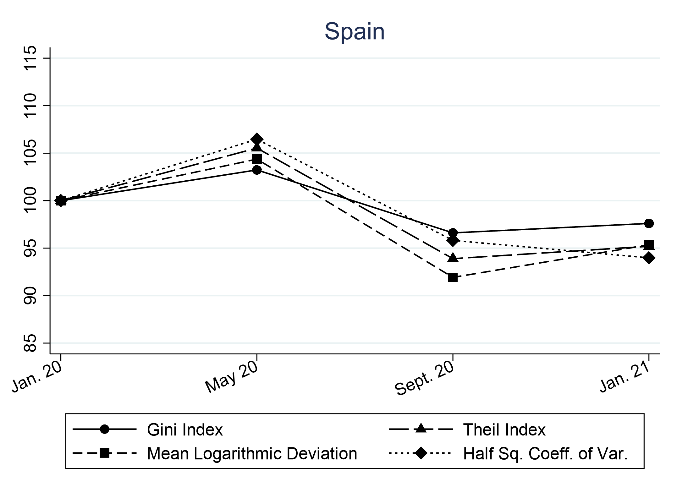


*Notes*. These figures refer to the analysis sample from the COME-HERE survey. “Income” refers to equivalised disposable household monthly income in Euros and PPP.

Figure A4: The evolution of Absolute Income Inequality in COME-HERE from January 2020 to January 2021 - Longitudinal Sample Weights


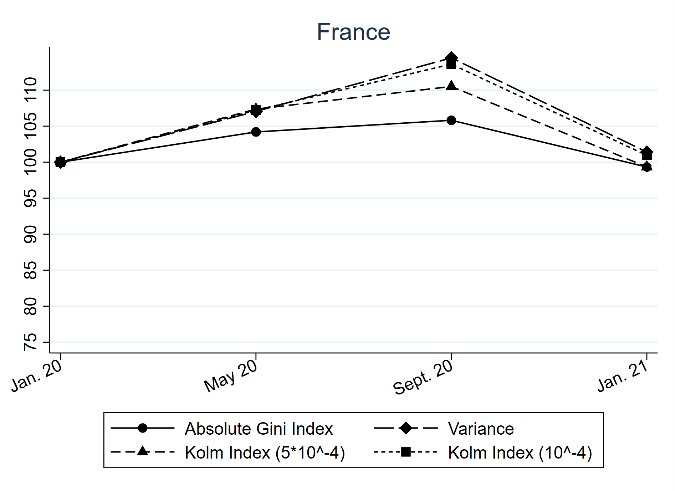

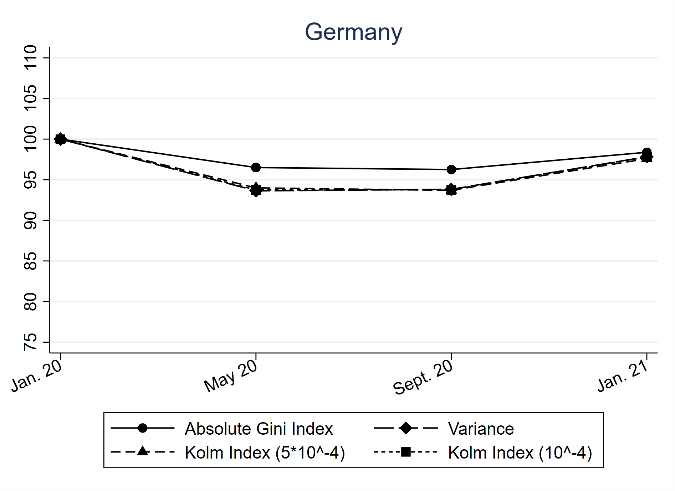

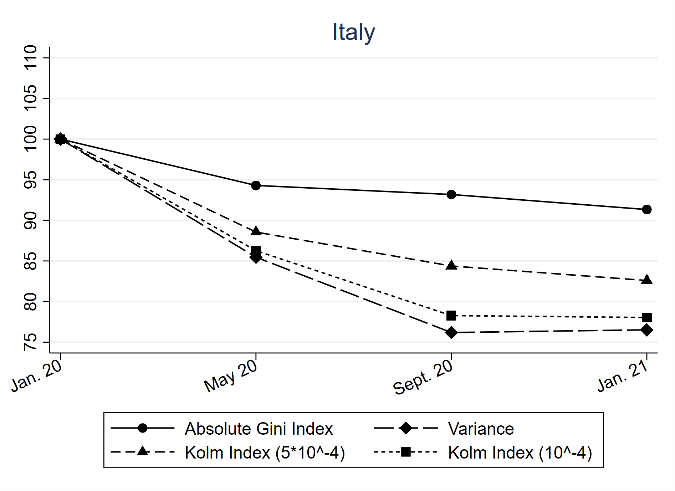

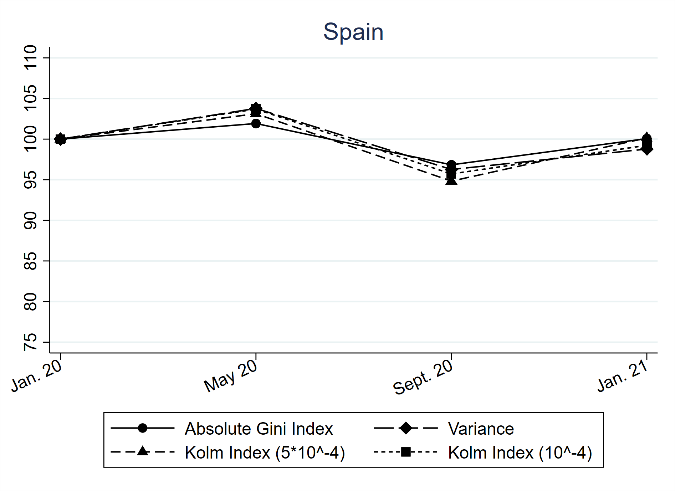


*Notes*. These figures refer to the analysis sample from the COME-HERE survey. “Income” refers to equivalised disposable household monthly income in Euros and PPP.

Figure A5: The evolution of Relative Income Inequality in COME-HERE from January 2020 to January 2021 – Split-histogram technique


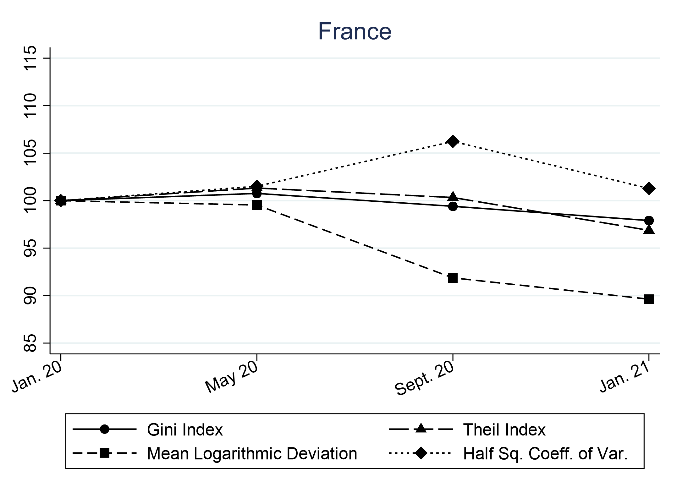

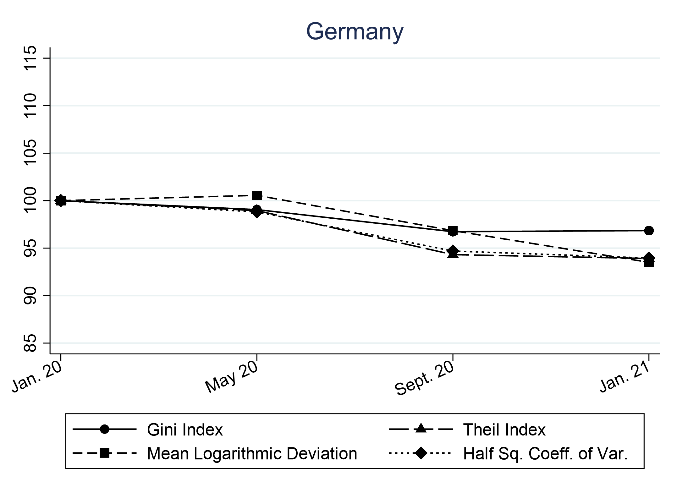

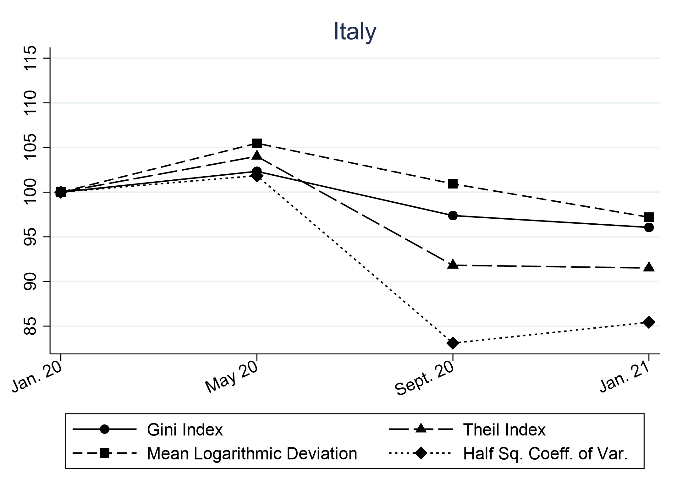

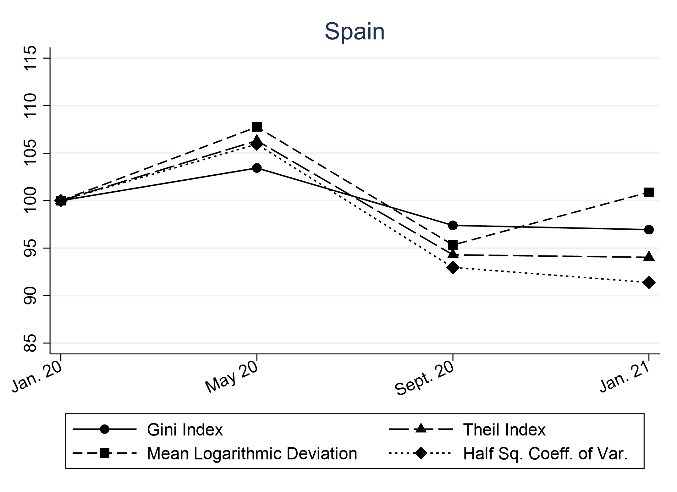


*Notes*. These figures refer to the analysis sample from the COME-HERE survey. “Income” refers to equivalised disposable household monthly income in Euros and PPP.

Figure A6: The evolution of Absolute Income Inequality in COME-HERE from January 2020 to January 2021 – Split-histogram technique


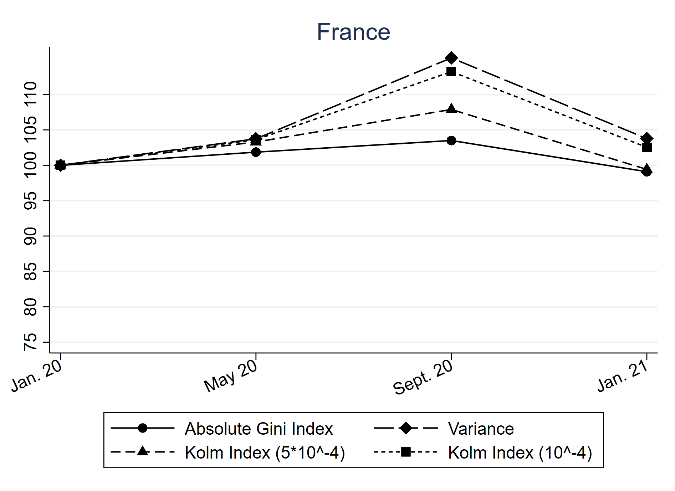

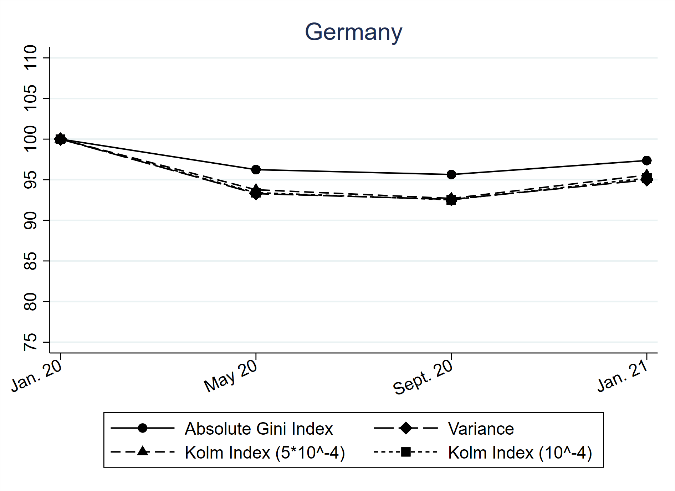

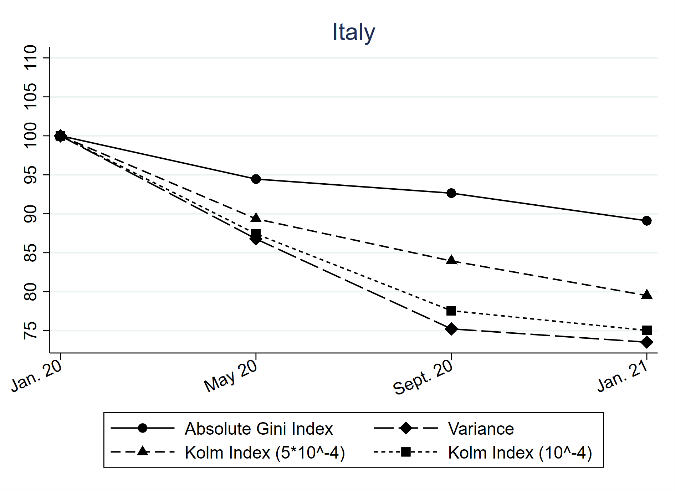

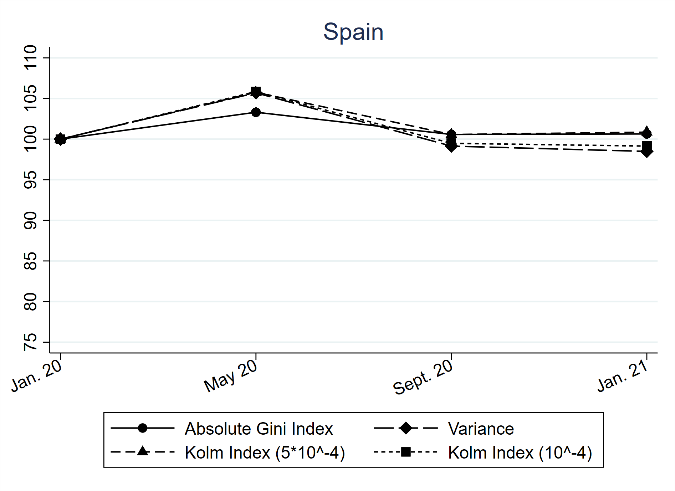


*Notes*. These figures refer to the analysis sample from the COME-HERE survey. “Income” refers to equivalised disposable household monthly income in Euros and PPP.

Figure A7: The evolution of Relative Income Inequality in COME-HERE from January 2020 to January 2021 – Considering within-band income changes


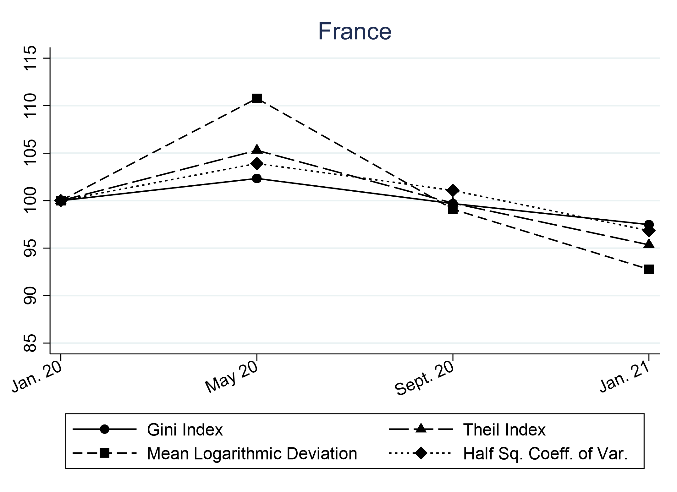

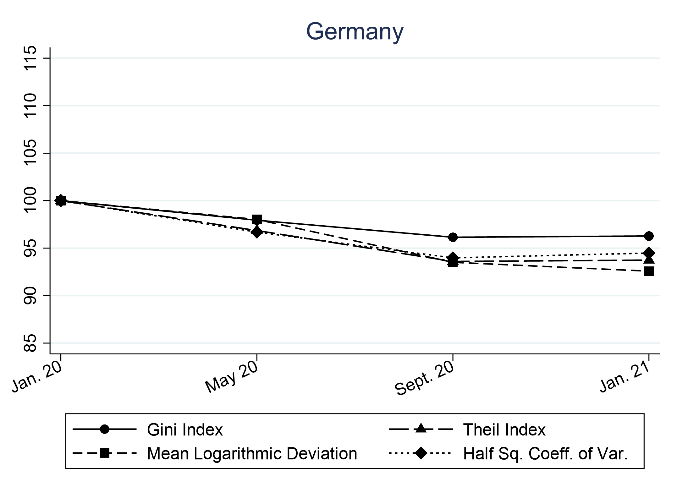

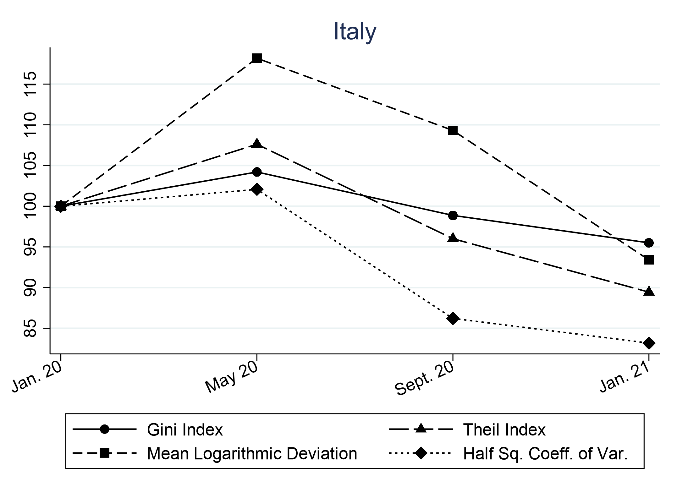

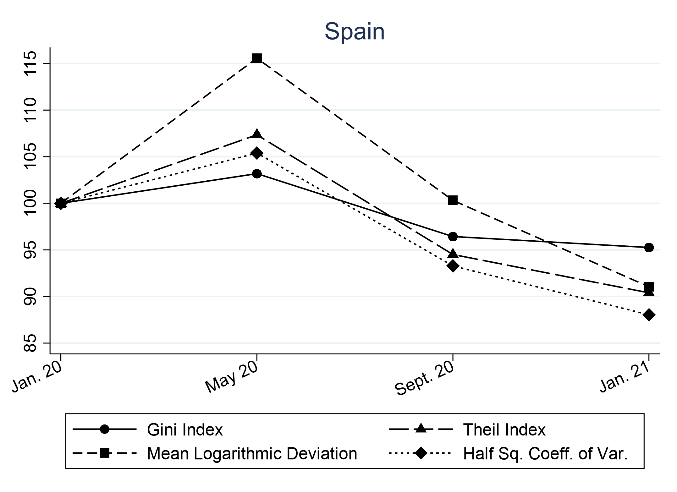


*Notes*. These figures refer to the analysis sample from the COME-HERE survey. “Income” refers to equivalised disposable household monthly income in Euros and PPP.

Figure A8: The evolution of Absolute Income Inequality in COME-HERE from January 2020 to January 2021 – Considering within-band income changes


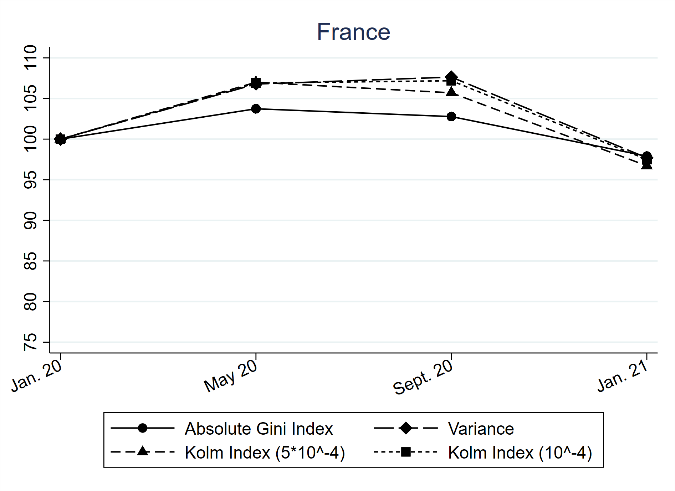

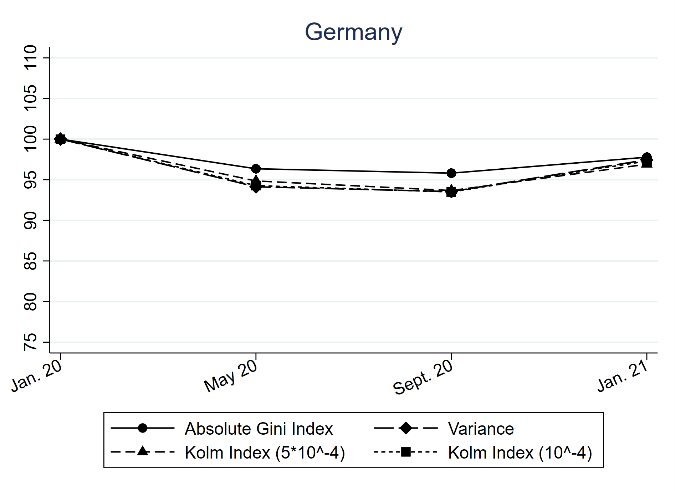

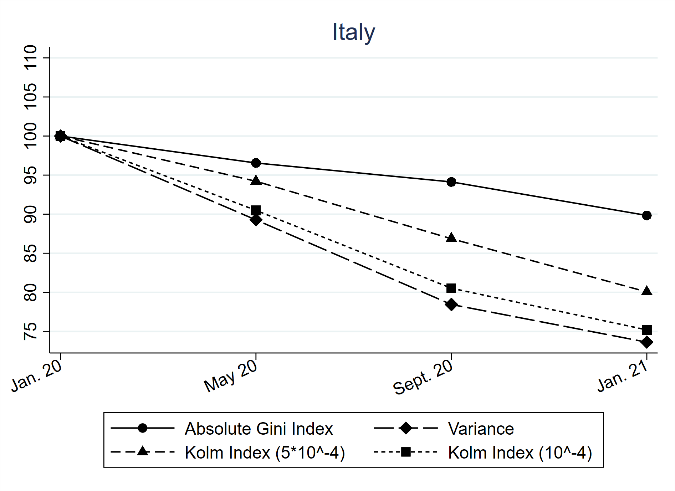

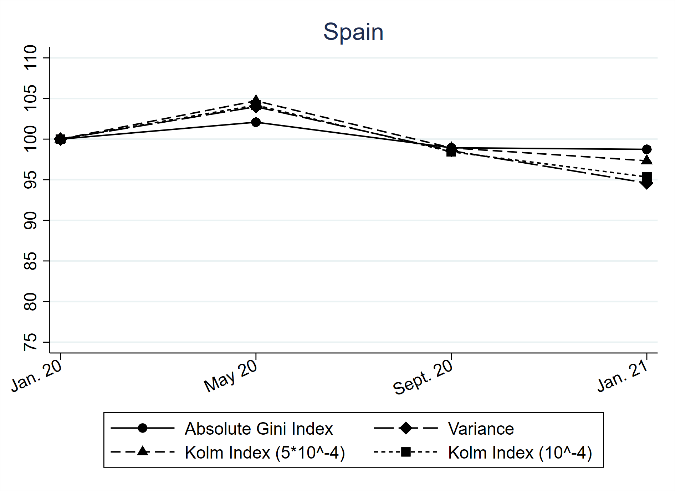


*Notes*. These figures refer to the analysis sample from the COME-HERE survey. “Income” refers to equivalised disposable household monthly income in Euros and PPP.
